# Integration of three-dimensional spinal traction and conventional rehabilitation for structural correction in adolescent idiopathic scoliosis: a case report

**DOI:** 10.3389/fspor.2026.1794187

**Published:** 2026-03-31

**Authors:** Ziheng Mao, Chenxi Li, Shugeng Chen, Zhiyang Zhao

**Affiliations:** 1 ASPINE Xuyang Doctor Group Co. Ltd., Shanghai, China; 2 Department of Rehabilitation Medicine, Shanghai Yuhua Rehabilitation Hospital, Shanghai, China; 3 Department of Rehabilitation Medicine, Huashan Hospital, Fudan University, Shanghai, China; 4 ASPINE Health Group Inc., Hayward, CA, United States

**Keywords:** adolescent, Cobb angle, idiopathic scoliosis, postural-based spinal rehabilitation, structural correction

## Abstract

**Background:**

This study investigated the improvement in sagittal and coronal plane posture in a female patient with adolescent idiopathic scoliosis (AIS) and associated pain symptoms, following a multidisciplinary comprehensive therapeutic intervention.

Case Presentation: The patient was a 12-year-old female who presented with several months of low back pain. A full-spine x-ray examination revealed cervical kyphosis, forward head posture (FHP), uneven shoulder height (with the left shoulder higher than the right), and a right-convex thoracolumbar scoliosis with a Cobb angle of 40°. The treatment utilized an innovative postural-based spinal rehabilitation, (PSR) which integrated various modalities such as postural correction training, muscle balance training, three-dimensional spinal traction (spinal 3D traction), and spinal manipulative therapy.

**Results:**

After 50 sessions over 32 weeks, the patient's postural deformity has showed marked improvement. FHP markedly decreased, thoracic positioning became more centered, shoulder leveling was restored, the right-convex thoracolumbar scoliosis Cobb angle decreased by 7° in the thoracic region and 17° in the lumbo-sacral region, the spinal rotation angle improved, and low back pain was greatly alleviated.

**Conclusion:**

Postural deformities, including adolescent idiopathic scoliosis, can be effectively corrected through a multidisciplinary comprehensive a treatment plan based on PSR, combined with conventional physical rehabilitation training.

## 1 Introduction

Scoliosis is a complex three-dimensional spinal deformity characterized by a lateral curvature in the coronal plane (Cobb angle ≥10°), an abnormal physiological curvature in the sagittal plane, and vertebral axial rotation. Clinically, scoliosis is classified based on its etiology into idiopathic and non-idiopathic types. Adolescent Idiopathic Scoliosis (AIS) is the most common among idiopathic cases; non-idiopathic types include congenital, neuromuscular, syndromic (e.g., Marfan syndrome), and secondary (e.g., trauma, tumor) subtypes (1, 2).

AIS typically occurs during the peak growth period of 10–18 years, with a global prevalence of 1.5%–3.0% and a notable higher incidence in females than males (approximately 3:1). The pathogenesis of AIS involves multifactorial interactions, including genetic predisposition (e.g., SNP mutations), asymmetric spinal growth (abnormal vertebral epiphyseal plate regulation), and neuromuscular control imbalance (proprioceptive deficits and abnormal muscle tone). This condition not only causes postural deformity and back pain but may also lead to progressive cardiopulmonary limitations (e.g., a 30% reduction in vital capacity when Cobb angle >40°) and psychological comorbidities such as anxiety and depression, severely impacting adolescents' quality of life. In actual clinical management of AIS, the proportion of patients undergoing surgery or long-term bracing remains relatively low (2). This clinical phenomenon may be explained by the following considerations. First, in adolescents with large curves who are at high risk of progression during peak growth, early conservative management is implemented to prevent progression to surgical thresholds. Such management typically consists of bracing combined with scoliosis-specific exercises (SSE), as recommended by current guidelines (3). Given the multiple unstable factors during adolescent growth, the potential for rapid curve progression, and the relatively higher surgical risks in this period, conservative management is generally prioritized before surgical intervention is considered. Intervention and improvement of scoliosis when patients cannot effectively adhere to traditional conservative treatments and bracing. Patients, due to demands for relatively high-intensity activities, were unable to comply with prescribed bracing duration. Although bracing is widely recognized as one of the most effective conservative strategies for preventing curve progression in AIS, patient compliance may be affected by discomfort or sleep disturbance in certain cases. In the present case, the patient reported increased nocturnal discomfort during bracing, which influenced adherence. It should be noted, however, that according to the SOSORT guidelines, bracing remains a cornerstone of conservative management for skeletally immature adolescents at risk of progression (2–4). Therefore, rather than replacing bracing, the present intervention was designed as a complementary conservative strategy combining posture-based spinal rehabilitation (PSR) with active exercise training and conventional rehabilitation techniques. This case provides preliminary insights into alternative conservative approaches that may improve symptom management and functional outcomes in selected patients. Further controlled studies are required to validate these findings (5, 6).

The case study involves a young female patient with Adolescent Idiopathic Scoliosis (AIS) and pain symptoms. Unable to tolerate bracing effectively and lacking subjective motivation for surgery, she opted for a novel regimen that combined PSR with conventional physical rehabilitation training, achieving significant improvement. This provides innovative and effective clinical guidance and demonstrates the partial feasibility of non-surgical, non-bracing corrective protocols for idiopathic scoliosis patients.

## 2 Case description

### 2.1 Case study

Female patient, age 12 years, presented on March 14, 2024, with uneven shoulder height and poor posture.

#### 2.1.1 Physical examination

**Figure 1:**
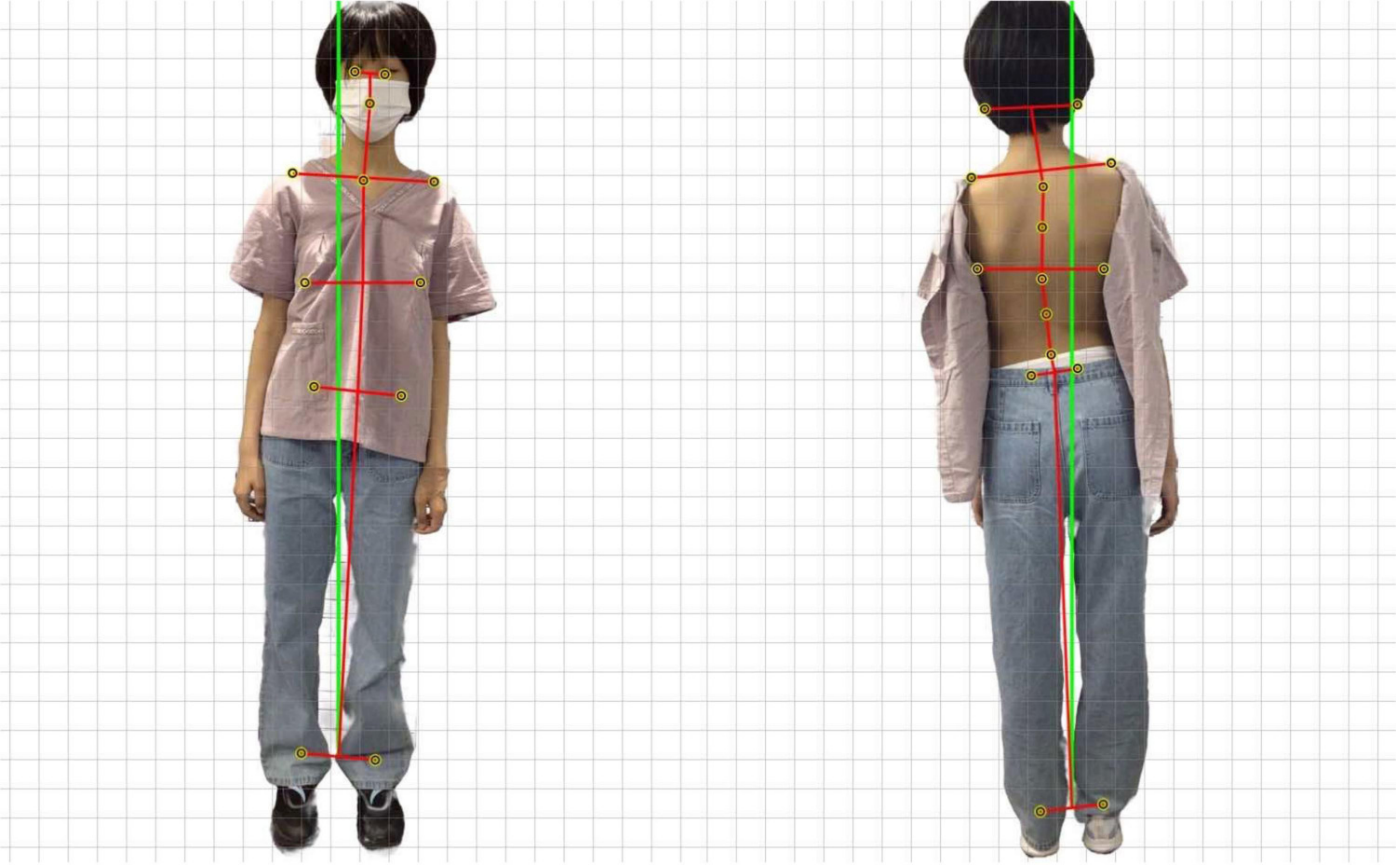
Right-tilted posture during initial assessment.

Uneven shoulders, rightward tilt; Anterior Superior Iliac Spine (ASIS) asymmetry (Rt+) visually measured; Right thoracic paraspinal muscle tension with press pain, left lumbar paraspinal muscle tenderness; Leg length discrepancy (Lt shorter, Lt(−)); Adam's Forward Bend Test (+); Scoliometer^©^ showed right rotation at T8 (7°) and left rotation at L2 (14°) (see Figure 1).

#### 2.1.2 Imaging examination

**Figure 2:**
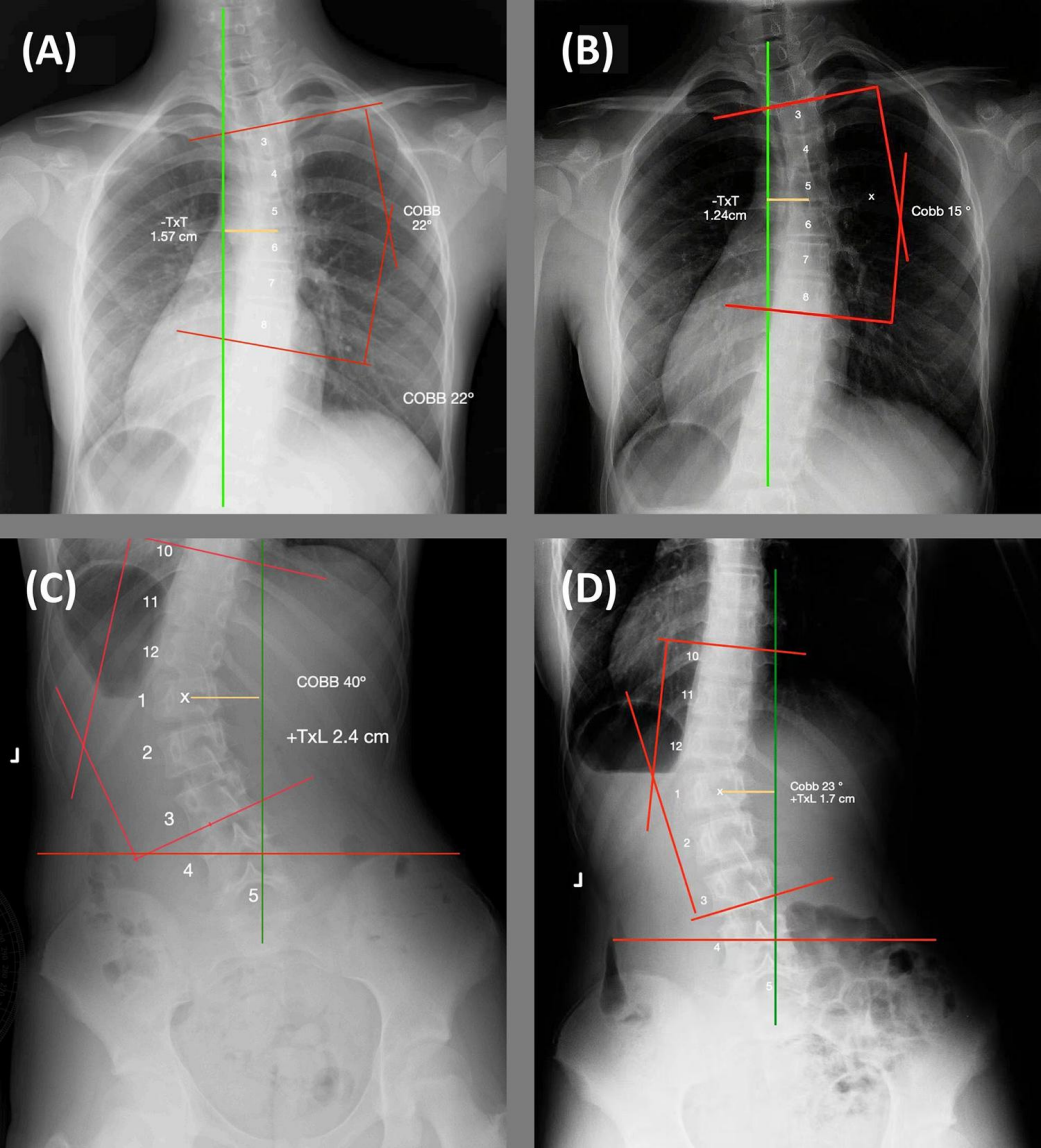
Comparison of initial x-rays and x-rays at 32-week assessment: **(A)** initial thoracic cobb angle 22° in x-ray; **(B)** initial lumbosacral cobb angle 40° in x-ray; **(C)** 32-week thoracic cobb angle 15° in x-ray; **(D)** 32-week lumbosacral cobb angle 23° in x-ray.

Thoracic x-ray (AP view) showed right convexity (−TxT) with rotation at T3–T8; Apex Vertebra Distance from Center Sacral Vertical Line (CSVL) 1.57 cm; ASPINE-specific AI analysis software measured Cobb angle 22.0°; Grade I vertebral rotation to the right (see Figure 2A). Lumbar x-ray (AP view) showed left convexity (+TxL) with rotation at T10-L3; Apex Vertebra Distance from CSVL 2.4 cm; ASPINE-specific AI analysis software measured Cobb angle 40.0°; Grade II vertebral rotation to the left (see Figure 2B).

### 2.2 Quantitative assessment

Multiple assessment tools were used to quantitatively evaluate the patient's pain level and functional improvement, while also attempting to exclude other potential influencing factors unrelated to the intervention.

Visual Analog Scale (VAS): Used for initial assessment of subjective pain intensity. Scores range from 0 to 10, where 10 represents “worst imaginable pain” or “most severe symptoms.” Its clinical significance lies in providing a rapid, intuitive quantification of subjective symptoms (e.g., pain, anxiety), facilitating monitoring of disease progression and treatment efficacy. Modified Ashworth Scale (MAS): Used for assessing muscle tone. Scores range from Grade 0 (no increase in muscle tone) to Grade 4 (affected part rigid). It is utilized to evaluate the severity of muscle tone during daily activities and movement. The clinical significance lies in the objective quantification of muscle tone status, which guides rehabilitation planning and outcome evaluation. In this instance, the MAS was introduced early in the intervention to quantify the level of muscle tone in the tender right thoracic and left lumbar paraspinal muscles (7, 8). Manual Muscle Testing (MMT): MMT scores typically range from Grade 0 (no contraction) to Grade 5 (normal strength). Used to assess asymmetry and weaknesses in the lumbosacral core muscle groups. Its clinical significance lies in guiding individualized rehabilitation program design to improve spinal stability and delay curve progression. Balance Error Scoring System (BESS): Used for bilateral plantar pressure imbalance caused by leg length discrepancy. Scores range from 0 to 60 (higher scores indicate more errors), assessing static balance function and fall risk. Applicable for balance screening in neurological patients and athletes (see Table 1).

**Table 1:** Assessment results at three time points.

Assessment tool	Pre-Tx assessment	16-week assessment	32-week assessment
VAS	4	3	2 (max 3 during exercise)
MAS	2	2	1+
MMT (Erector Spinae, Iliopsoas)	3+	4	4+
BESS	17	15	11
Cobb（app）	(T)22.0°	(T)23.0°	(T)15.0°
	(L)40.0°	(L)36.0°	(L)23.0°
Cobb（manual）	(T)22°	(T)22°	(T)18°
	(L)40°	(L)38°	(L)23°
Spinal Rotation Angle Thoracic (T)	(T)12°	(T)15°	(T)9°
	(L)27°	(L)20°	(L)15°

T, Thoracic,Max Rotation Angle; L, Lumbar,Max Rotation Angle.

**Figure 3:**
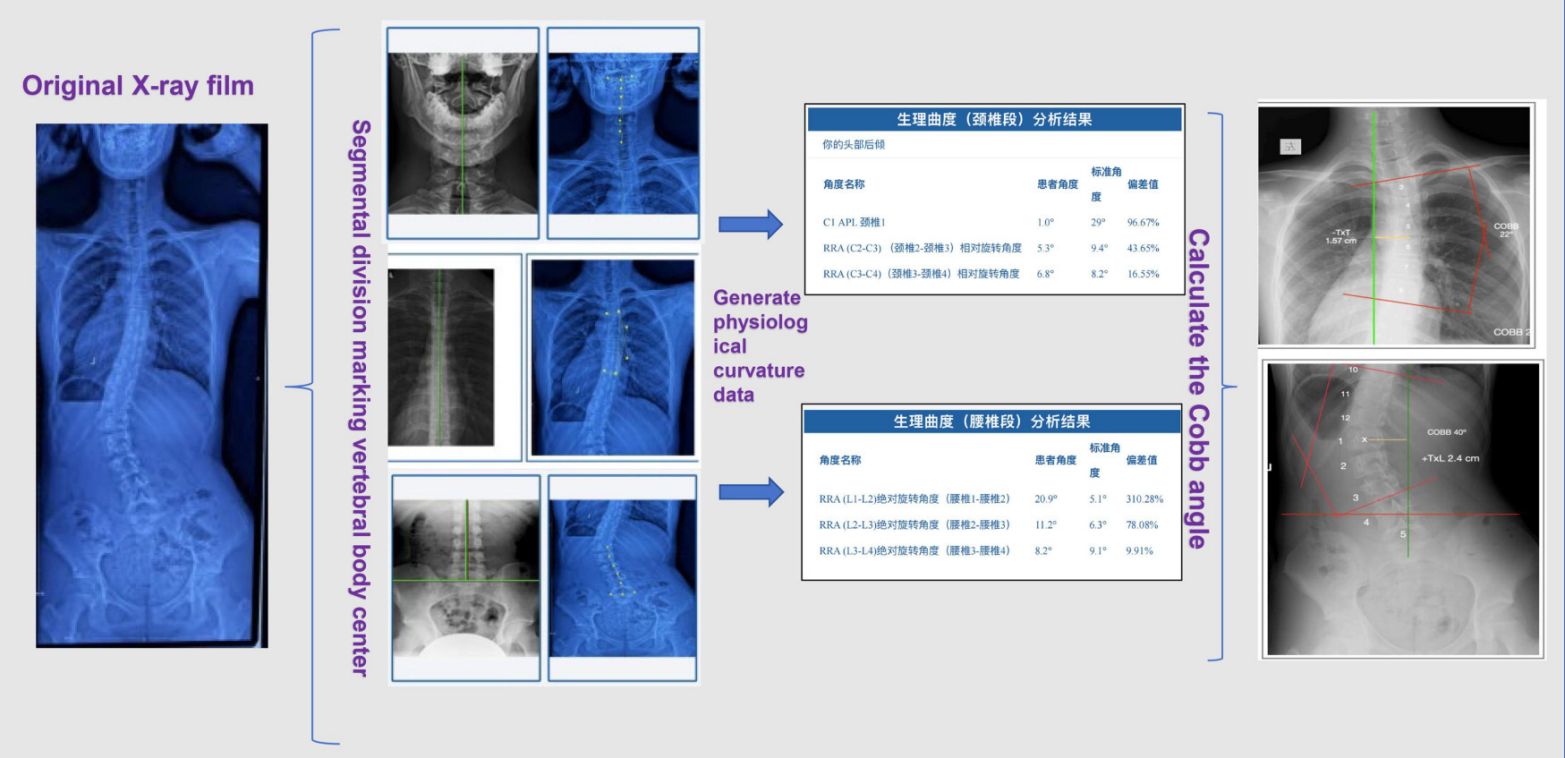
Program flowchart.

AI Algorithm-Assisted Application (App): This study utilized an AI algorithm-assisted software program that incorporates a computer-vision–based artificial intelligence module designed to automate the digitisation of radiographic images, particularly for lateral cervical and lumbar projections. This AI functionality performs automatic anatomical landmark identification and measurement, significantly reducing manual processing time while maintaining the requirement for clinician validation prior to clinical interpretation. The system operates through a proprietary cloud interface and has been implemented as an objective radiographic mensuration tool in chiropractic clinical research. In this application, the software was used to calculate Cobb angles and spinal rotation angles alongside manual measurements to evaluate overall scoliosis improvement (9, 10). The algorithm was trained on a dataset of 3,000–4,000 scoliosis x-rays to enable autonomous analysis, original radiograph were input into the software and relevant segments were cropped. The program established a baseline for the healthy spine segment; following the marking of vertebral midpoints, it calculated the physiological curvature deviation angle to generate the final Cobb angle result (11–13). (See Figure 3).

### 2.3 Rehabilitation goals

Maintain scoliosis Cobb angle below surgical threshold (<40°) through 50 sessions; Reduce spinal rotation angle and lateral curvature under PSR.

### 2.4 Treatment plan formulation

Based on full-spine x-ray analysis, this AIS patient presented with an S-shaped curve (left lumbar, right thoracic), with the primary curve located in the lumbar region accompanied by pelvic sacral base rightward deviation (Sacral Anterior Right, SAR). Therefore, the primary adjustment direction targeted the sacrum and lumbar spine using a reverse postural positioning (RPP) principle aimed at restoring neutral spinal alignment. 1. Full-spine adjustment using a Drop Table (DT). 2. Three-dimensional corrective traction was applied in combination with targeted postural corrective exercises; a gravity-assisted traction platform was constructed using standard therapeutic equipment to provide focused correction to the scoliotic segments.

### 2.5 Treatment implementation

This protocol employed a progressive “Pelvis First, Gradual Correction” adjustment strategy. This involved first restoring pelvic biomechanical stability through pelvic adjustment, followed by three-dimensional correction of the entire thoracolumbosacral segment based on a stable pelvis. Phase 1 focused on pelvic adjustment, utilizing gravity traction, core muscle activation, and RPP training to optimize pelvis-lumbar alignment and reduce mechanical compensation for scoliosis. Phase 2 involved integrated thoracolumbar adjustment, incorporating resistance training and dynamic correction techniques following enhanced pelvic stability to achieve synergistic correction of the thoracolumbar segment(see Table 2).

**Table 2:** Two-phase treatment plan.

Phase	Weeks	Therapeutic Goals	Core Therapeutic Content	Key Parameters
Pelvic Adjustment Period (1–12wks)	1–4	Pelvic biomechanical reconstruction; Reduce lumbar rotational stress	Gravity traction: Right side-lying, left hip flexed, sandbag fixation L1–L3 Right-side down left leg raise Cervical ROM training Core activation basics (plank)	15 min/session 10 s × 10 sets 20 reps 30 s × 3 sets
	5–8	Enhance pelvis-lumbar alignment	+TRX rhomboid row + squat integration +Swiss ball left-side stretch (convex side ES/concave side iliopsoas) +MI training (seated): Right rotation + right translation + left lateral flexion +Full spine stretch (pectorals)	3 sets × 15 reps 1 min × 3 sets 50 reps 5 sets × 7 s
	9–12	Pelvic stability reinforcement	Advanced gravity traction (increased duration) Resisted leg raise (right-side down) Enhanced MI training (yoga block assisted) Advanced TRX compound movements	20 min/session 15 s × 10 sets 80 reps 3 sets × 20 reps
Thoracolumbar Joint Adjustment Period (13–32wks)	13–20	Three-dimensional thoracolumbar correction	Resisted MI (standing): Pelvis fixed with elastic band, foam pad limiting Upgraded gravity traction (L1–L3 fixation) Swiss ball left-side core training I/Y/W alphabet training	Right rot + trans + lat flex × 50 reps 25 min/session 2 min × 5 sets 10 sets
	21–28	Redistribution of abnormal muscle tone	+Elastic band resisted 3D correction (Right rot + trans + lat flex) +Advanced plank +Increased load TRX row + squat +Multi-directional full spine stretch	100 reps × 1 set 1 min × 3 sets 3 sets × 25 reps 5 sets × 10 s
	29–32	Functional integration & consolidation	Compound resisted MI (80–100° dynamic correction) Unstable surface training (Swiss ball) ADL simulation integration training 3D correction endurance training	100 reps × 1 set 3 min × 3 sets Specially designed 5 sets × 20 s

#### 2.5.1 Pelvic focus

**Figure 4:**
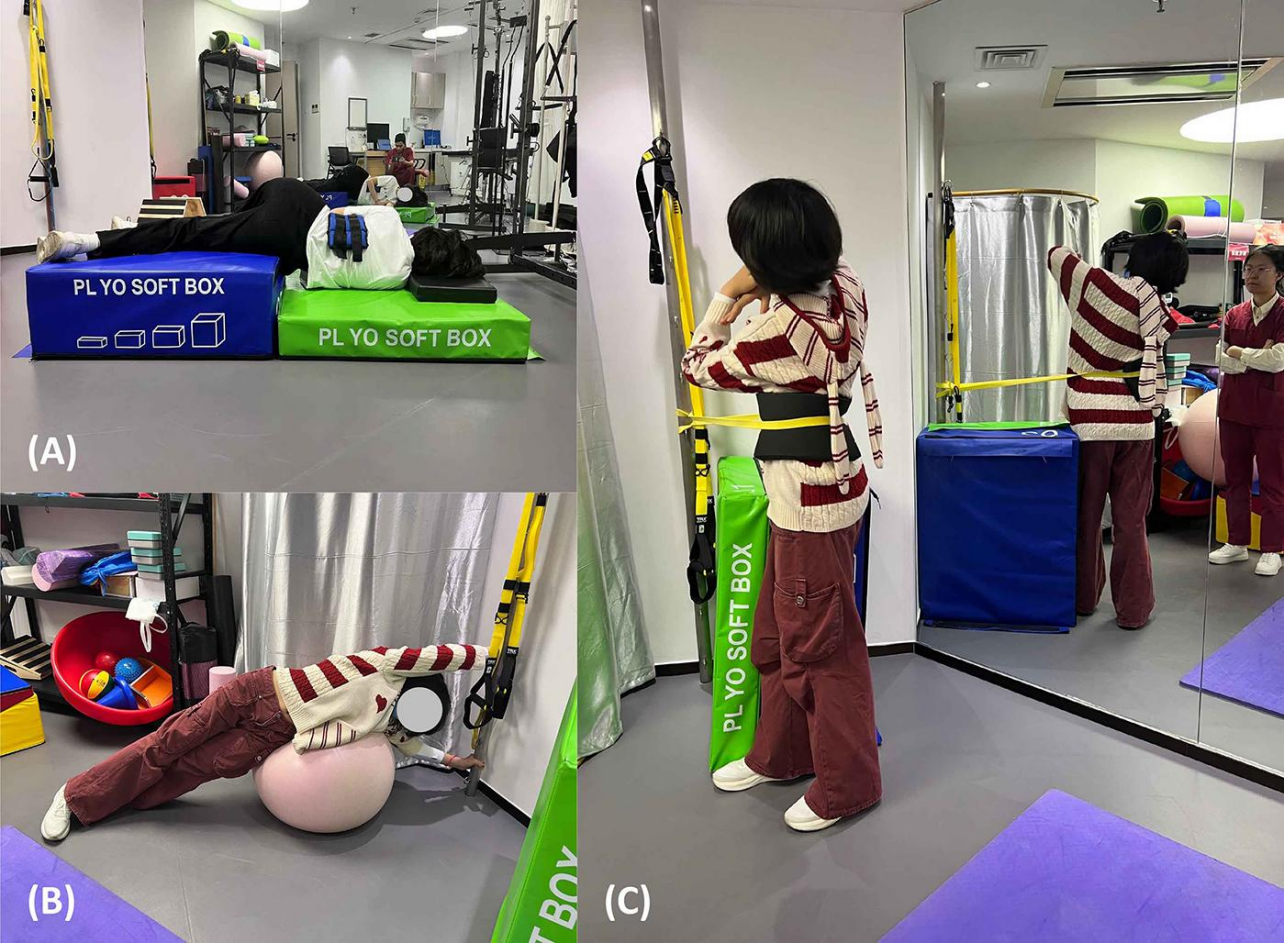
Three types of training scenarios. **(A)** Gravity traction Training; **(B)** Swiss Ball Training; **(C)** Mirror Image Training.

Cervical ROM training ×20 reps; Gravity traction: Right side-lying, left hip flexed, sandbag fixation L1–L3 aimed at reducing lumbar rotational stress via axial traction, while the left hip flexed position enhanced eccentric contraction of the convex side (right) iliopsoas to promote pelvic symmetry restoration (see Figure 4A); Followed by right-side down left leg raise 10 s × 10 sets, rest 10 min; Total Resistance Exercise (TRX) rhomboid row training + squat ×20 reps; Swiss ball left-side stretch (convex side ES/concave side iliopsoas) for core strength (see Figure 4B); MI training: Patient seated, left foot on yoga block, performing right rotation + right translation + left lateral flexion ×50 reps (see Figure 4C); Full spine stretch (pectorals) 5 sets × 7 s

#### 2.5.2 Thoracolumbar joint adjustment

Cervical ROM training ×30 reps. Gravity traction: Right side-lying, left hip flexed, sandbag fixation L1–L3, hold 15 min, then right-side down left leg raise 10 s × 10 sets, rest 10 min; TRX rhomboid row training + squat ×20 reps; Swiss ball left-side down 1 min (stretch convex ES/concave iliopsoas, train core strength); Resisted MI: Patient standing, foam pad placed under left 12th rib to limit non-target segment motion, elastic band stabilizing pelvis, guiding active resisted right rotation + translation + left lateral flexion (see Figure 4C), strengthening symmetrical spinal movement patterns and correcting abnormal muscle tone distribution; Plank 1 min ×  3 sets; I/Y/W exercises 10 sets; Right rotation + right translation + left lateral flexion ×50 reps; Full spine stretch (pectorals) 5 sets ×7 s

#### 2.5.3 Lifestyle recommendations

Perform stretching and warm-up exercises before and after any activity; keep mobile devices at eye level to avoid forward head posture; avoid sitting for more than one hour continuously during home or work periods, and take breaks every 30–40 min.

## 3 Results

After 50 sessions spanning 32 weeks, the patient exhibited a notable reduction in VAS score, a decrease in muscle tone, substantial improvement in the strength of the erector- spinae and iliopsoas muscles (see Table 1). The thoracic Cobb angle diminished by 7 degrees and the spinal rotation angle by 3 degrees; the lumbosacral Cobb angle decreased by 17 degrees and the spinal rotation angle by 12 degrees, moving the patient further from the surgical threshold (Cobb >40°). These outcomes support the potential of PSR as an alternative to bracing. Radiographic improvement was demonstrated by the reduced Cobb angles in follow-up full-spine AP x-rays (see Figures 2C,D), indicating the treatment's efficacy.

## 4 Discussion

After 50 sessions of combined PSR, 3D traction, and RPP intervention, this study demonstrated significant improvements in the Cobb angle and vertebral rotation angle for a patient with severe AIS who was intolerant to braces and refused surgical intervention. This outcome offers a new perspective in conservative management, particularly addressing a treatment gap for special populations, such as those allergic to braces, psychologically resistant, or facing economic constraints (9, 14, 15).

The “ASPINE systems protocol + active training” strategy aligns closely with the internationally advocated multimodal intervention concept of “bracing + specific exercises.” Schroth exercises combined with Chêneau bracing have been confirmed by multiple Randomized Controlled Trials (RCTs) to reduce curve progression risk (success rate 83.2%) and significantly improve quality of life (SRS-22 score increase of 1.41 points) (10). Similarly, this protocol achieved precise exercise customization through software-assisted dynamic Cobb angle calculation, echoing the international trend towards individualized treatment. For instance, the Gensingen brace (GBW), constructed using CAD/CAM technology for patient-specific orthotic cavities, shows significantly higher success rates (>90%) than traditional symmetric braces (72%), confirming the core role of individualized biomechanical intervention (16–18).

Distinct from mainstream bracing-dependent protocols for severe curves, this study innovatively provided a feasible alternative for brace-intolerant individuals through an active correction combination of “3D traction + MI training.” This approach complements the application of Schroth exercises in mild-moderate curves (e.g., Cobb reduction from 30.6° to 18.5° in a 25-year-old (14, 15, 19)) but is novel in demonstrating the effectiveness of a non-surgical, non-bracing protocol in a brace-contraindicated severe patient (Cobb >40°) (14).

Regarding the mechanism, unlike traditional bracing that relies on static mechanical inhibition, this protocol employs Motor Imagery (MI) training to achieve dynamic three-dimensional correction through neuromuscular re-education (e.g., resisted right rotation, translation, and left lateral flexion). While this design approximates the Schroth “corrective breathing” concept (20), it innovatively incorporates software real-time feedback to optimize exercise precision. The core innovations of this study lie in two technological breakthroughs: the software-assisted quantitative assessment system and the functional integration correction pathway.

Dynamic calculation of Cobb and rotation angles via imaging analysis software overcomes the limitations of traditional surface measurements (e.g., Scoliometer^©^) affected by soft tissue interference (17). Similar in value to Finite Element Analysis (FEA) for brace optimization, this approach extends into the active training domain, providing immediate data support for exercise adjustments and avoiding the correction efficacy decay seen in traditional Schroth exercises due to subjective postural perception bias (14, 17).

Integrating structural spinal manipulation, 3D traction (spatial alignment), and RPP training (neural control) forms a “Structure-Space-Function” three-tier correction chain. Previous studies have reported average Cobb angle improvements of approximately 3–4° following single-modality Schroth exercises, which may approach the margin of measurement variability (2). This study achieved multi-dimensional improvement simultaneously in a severe case, suggesting a potential integrative benefit that warrants further investigation (7). The significant clinical implication of this study is providing a new intervention option for three populations: brace-intolerant individuals (skin allergy, psychological resistance), those hesitant about surgery or economically constrained, and adolescents with remaining growth potential.

As a single-case report, this study lacks broader evidence-based support. Generalization of results requires validation through large-sample Randomized Controlled Trials (RCTs) (21, 22). Much assessment data originated from a single subject, making it difficult to control variables, and results are susceptible to external confounding factors. Additionally, the treatment frequency (50 sessions) may impose high economic and time costs. Based on these limitations, future research should prioritize RCT validation.

## 5 Conclusion

Through a detailed case analysis of an adolescent patient, this report demonstrates the efficacy of conservative management for idiopathic scoliosis, emphasizing the importance and necessity of non-surgical approaches within this patient population. The case illustrates that PSR, when combined with conventional physical rehabilitation training, is a genuinely effective intervention modality in the rehabilitation of idiopathic scoliosis. The study results provide valuable references for clinical practice, offering a novel and effective therapeutic approach for patients who cannot tolerate bracing effectively and lack subjective motivation for surgery.

## Data Availability

The raw data supporting the conclusions of this article will be made available by the authors, without undue reservation.

## References

[1] Charalampidis A Diarbakerli E Dufvenberg M Jalalpour K Ohlin A Ahl AA Nighttime bracing or exercise in Moderate-Grade adolescent idiopathic scoliosis. JAMA Network Open. (2024) 7(1):e2352492. 10.1001/jamanetworkopen.2023.52492 38285447 PMC10825714

[2] Dolan LA Weinstein SL Abel MF Bosch PP Dobbs MB Farber TO Bracing in adolescent idiopathic scoliosis trial (BrAIST): development and validation of a prognostic model in untreated adolescent idiopathic scoliosis using the simplified skeletal maturity system. Spine Deform. (2019) 7(6):890–8.e4. 10.1016/j.jspd.2019.01.011 31731999 PMC6939758

[3] Negrini S Donzelli S Aulisa AG Czaprowski D Schreiber S de Mauroy JC 2016 SOSORT guidelines: orthopaedic and rehabilitation treatment of idiopathic scoliosis during growth. Scoliosis Spinal Disord. (2018) 13:3. 10.1186/s13013-017-0145-8 29435499 PMC5795289

[4] Mohammed AH Abo-Ali SE Abdelmutilibe SM Elsamahy SA Elsherif NE Elmahdy MA Muscle energy techniques versus myofascial release on scoliosis in adolescent girls: a randomized controlled trial. Fizjoterapia Polska. (2024) 24(1):54–9. 10.56984/8ZG2EF864D

[5] Guo J. Intervention and correction of scoliosis through dance body training. AdvPhys Sci. (2023) 11(4):1155–1162. 10.12677/APS.2023.114159.

[6] Oakley PA Harrison DD Harrison DE Haas JW. Evidence-based protocol for structural rehabilitation of the spine and posture: review of clinical biomechanics of posture (CBP) publications. J Can Chiropr Assoc. (2005) 49(4):270–296. PMCID: PMC1840024. 17549209 PMC1840024

[7] Schreiber S Parent EC Moez EK Hedden DM Hill DL Moreau M Schroth physiotherapeutic scoliosis-specific exercises added to the standard of care lead to better cobb angle outcomes in adolescents with idiopathic scoliosis—an assessor and statistician blinded randomized controlled trial. PLoS One. (2016) 11(12):e0168746. 10.1371/journal.pone.0168746 28033399 PMC5198985

[8] Lonstein JE. Idiopathic scoliosis. In: Morrissy RT Weinstein SL, editors. Lovell and Winter’s Pediatric Orthopaedics. 6th ed. Philadelphia: Lippincott Williams & Wilkins (2006). p. 694–762.

[9] Maaliw RR. SCOLIONET: an automated scoliosis Cobb angle quantification using enhanced X-ray images and deep learning models. J Imaging. (2023) 9(12):265. 10.3390/jimaging9120265 38132683 PMC10743962

[10] Hosseini MM Mahoor MH Haas JW Ferrantelli JR Dupuis A-L Jaeger JO Intra-Examiner Reliability and Validity of Sagittal Cervical Spine Mensuration Methods Using Deep Convolutional Neural Networks. J Clin Med. (2024) 13(9):2573. 10.3390/jcm13092573. 38731102 PMC11084751

[11] Longo K Haas JW Oakley PA Harrison DE. Reduction in severe chronic mid-back pain following Chiropractic BioPhysics® rehabilitation with corresponding radiographic changes: a case report. Healthcare. (2025) 13(20):2587. 10.3390/healthcare13202587 41154265 PMC12564805

[12] Harrison DE Harrison DD Oakley PA Haas JW Betz JW DeBord D Computerized radiographic digitization for the quantification of spinal biomechanical parameters using PostureRay®. Available online at: https://www.researchgate.net/ (Accessed December 10, 2025).

[13] Harrison DE Oakley PA Harrison DD. Scoliosis deformity reduction in adults: a CBP® Mirror Image® case series incorporating the non-commutative property of finite rotation angles under addition. J Phys Ther Sci. (2017) 29:2062–7. 10.1589/jpts.29.2044 29200654 PMC5702844

[14] Moustafa I Youssef ASA Ahbouch A Harrison D. Demonstration of autonomic nervous function and cervical sensorimotor control after cervical lordosis rehabilitation: a randomized controlled trial. J Athl Train. (2021) 56(4):427–36. 10.4085/1062-6050-0481.19 33543266 PMC8063661

[15] Moustafa IM Diab AAM Shima T Harrison DE. The effect of normalizing the sagittal cervical configuration for the management of cervicogenic headaches: a 2- year pilot randomized controlled trial. Proceedings of the 15th Biennial Congress of the World Federation of Chiropractic (2019).

[16] Chan WWY Fu S-N Chong T-F Singh G Tsai DSJ Wong MCY Associations between paraspinal muscle characteristics and spinal curvature in conservatively treated adolescent idiopathic scoliosis: a systematic review. Spine J. (2024) 24(4):692–720. 10.1016/j.spinee.2023.11.008 38008187

[17] Wong AYL Effectiveness of acceptance and commitment therapy for parents and children with adolescent idiopathic scoliosis: a randomized controlled trial. ClinicalTrials.gov NCT05919459. (2024). Available online at: https://ichgcp.net/zh/clinical-trials-registry/NCT05919459 (Accessed June 05, 2025)

[18] Razeghinezhad R Kamyab M Babaee T Ganjavian MS Bidari S. The effect of brace treatment on large curves of 40° to 55° in adolescents with idiopathic scoliosis who have avoided surgery: a retrospective cohort study. Neurospine. (2021) 18(3):437–44. 10.14245/ns.2040654.327 34634198 PMC8497257

[19] Chen C Xu J Li H. Effects of schroth 3D exercise on adolescent idiopathic scoliosis: a systematic review and meta-analysis. Children. (2024) 11(7):806. 10.3390/children11070806 39062255 PMC11275065

[20] Nan X Kuru Çolak T Akçay B Xie H Zhao L Borysov M. Results of Gensingen bracing in patients with adolescent idiopathic scoliosis: retrospective cross-sectional feasibility study. JMIR Rehabil Assist Technol. (2024) 11:e50299. 10.2196/50299 38198197 PMC10809064

[21] Weinstein SL Dolan LA Wright JG Dobbs MB. Effects of bracing in adolescents with idiopathic scoliosis. N Engl J Med. (2013) 369(16):1512–21. 10.1056/NEJMoa1307337 24047455 PMC3913566

[22] Huijberts I Pinckaers FME Olthuis SGH van Kuijk SMJ Postma AA Boogaarts HD Collateral-based selection for endovascular treatment of acute ischaemic stroke in the late window (MR CLEAN-LATE): 2-year follow-up of a phase 3, multicentre, open-label, randomised controlled trial in The Netherlands. Lancet Neurol. (2024) 23(9):893–900. 10.1016/S1474-4422(24)00228-X 38909624

